# Comparative molecular cytogenetic characterization of seven *Deschampsia (Poaceae)* species

**DOI:** 10.1371/journal.pone.0175760

**Published:** 2017-04-13

**Authors:** Alexandra V. Amosova, Nadezhda L. Bolsheva, Svyatoslav A. Zoshchuk, Maryana O. Twardovska, Olga Yu Yurkevich, Igor O. Andreev, Tatiana E. Samatadze, Ekaterina D. Badaeva, Viktor A. Kunakh, Olga V. Muravenko

**Affiliations:** 1 Engelhardt Institute of Molecular Biology, Russian Academy of Sciences, Moscow, Russian Federation; 2 Institute of Molecular Biology and Genetics, National Academy of Sciences of Ukraine, Kyiv, Ukraine; Huazhong University of Science and Technology, CHINA

## Abstract

The genus *Deschampsia* P. Beauv (*Poaceae*) involves a group of widespread polymorphic species. Some of them are highly tolerant to stressful and variable environmental conditions, and *D*. *antarctica* is one of the only two vascular plants growing in Antarctic. This species is a source of useful for selection traits and a valuable model for studying an environmental stress tolerance in plants. Genome diversity and comparative chromosomal phylogeny within the genus have not been studied yet as karyotypes of most *Deschampsia* species are poorly investigated. We firstly conducted a comparative molecular cytogenetic analysis of *D*. *antarctica* (Antarctic Peninsula) and related species from various localities (*D*. *cespitosa*, *D*. *danthonioides*, *D*. *elongata*, *D*. *flexuosa* (= *Avenella flexuosa*), *D*. *parvula* and *D*. *sukatschewii* by fluorescence *in situ* hybridization with 45S and 5S rDNA, DAPI-banding and sequential rapid *in situ* hybridization with genomic DNA of *D*. *antarctica*, *D*. *cespitosa*, and *D*. *flexuosa*. Based on patterns of distribution of the examined markers, chromosomes of the studied species were identified. Within these species, common features as well as species peculiarities in their karyotypic structure and chromosomal distribution of molecular cytogenetic markers were characterized. Different chromosomal rearrangements were detected in *D*. *antarctica*, *D*. *flexuosa*, *D*. *elongata* and *D*. *sukatschewii*. In karyotypes of *D*. *antarctica*, *D*. *cespitosa*, *D*. *elongata* and *D*. *sukatschewii*, 0–3 B chromosomes possessed distinct DAPI-bands were observed. Our findings suggest that the genome evolution of the genus *Deschampsia* involved polyploidy and also different chromosomal rearrangements. The obtained results will help clarify the relationships within the genus *Deschampsia*, and can be a basis for the further genetic and biotechnological studies as well as for selection of plants tolerant to extreme habitats.

## Introduction

The genus *Deschampsia* P. Beauv. (*Poaceae*) includes more than 30 polymorphic species with a wide geographical distribution, high morphological diversity and complicated taxonomy [1–6]. Some of *Deschampsia* species are highly tolerant to stressful and variable environmental conditions, and this tolerance allows them to colonize plots of land which are uninhabited by other plants [7]. The core of the genus is mainly represented by subspecies of nearly cosmopolitan *D*. *cespitosa* (L.) P. Beauv. (*D*. *cespitosa* complex) that can exist even in extreme Arctic habitats [8]. Compared to the Arctic flora with many flowering species, only two vascular plants were found in the Antarctic regions of the similar latitude, and *D*. *antarctica* E. Desv. is the only species in the *Poaceae* family (and the only *Deschampsia* species) that has developed morphological and physiological adaptation to the harshest Antarctic environments (extremely low temperatures, drought, high salinity and flooding, high level of UV radiation, low precipitation) [9–10]. *D*. *antarctica* could serve as a model for the study of regulation of genome activity and the mechanisms responsible for plant adaptation to freezing, light stress or photosynthetic capacity at low temperatures [11–16]. *D*. *antarctica* might also be a useful source of genes associated with stress tolerance and environmental adaptation, and its karyotype can be a basic tool for crop breeding strategies in agronomical valuable crops [17–19]. Besides, extracts from *D*. *antarctica* display protective effects against ultraviolet radiation and are suitable for a number of pharmaceutical applications [20].

The unique genome of *D*. *antarctica* is being intensively investigated. Particularly, a multi-gene family encoding ice recrystallization inhibition proteins which are related to freeze tolerance has been analyzed [15–16]; the chloroplast genome has been sequenced and plastid transcriptome profiles of the coding/noncoding genes have been studied [5, 18]; the polypeptide with lipase activity (Lip3F9) has been characterized [21]. However, currently insufficient nuclear genomic data is still available for this species [5, 16, 21].

Phylogenetic relationships within the genus *Deschampsia* is still being under investigation [22–23]. According to the phylogenetic studies based on the analysis of nuclear ribosomal internal transcribed spacer (ITS) and plastid trnL intron sequences, most *Deschampsia* species are joined in one clade. Inside this clade, *D*. *antarctica* together with *D*. *venustula* Parodi and *D*. *parvula* (Hook.f.) E. Desv. forms a small subclade. It was suggested that two species *D*. *antropurpurea* (Wahlenb.) Scheele (= *Vahlodea atropurpurea* (Wahlenb.) Fr. and *D*. *flexuosa* (L.) Trin. (= *Avenella flexuosa* (L.) Drejer)) should be excluded from the genus *Deschampsia* and included to the allied genera *Vahlodea* and *Avenella* (correspondingly) despite morphological similarity of *D*. *flexuosa* and *D*. *cespitosa* [2, 22–24].

The analysis of karyotypes of *Deschampsia* species performed by modern molecular cytogenetic methods is important for better understanding of phylogeny, taxonomy and evolution of the genus and for investigation of the unique *D*. *antarctica* genome. However, genome diversity and comparative chromosomal phylogeny in the genus *Deschampsia* have not been studied as currently available molecular cytogenetic data on most *Deschampsia* species are rather limited. In three species, *D*. *cespitosa*, *D*. *flexuosa* and *D*. *setacea* (Huds.) Hack., distribution of Giemsa C-heterochromatin was described [1]. In *D*. *cespitosa*, chromosomal localization of rDNA and satellite DNAs was detected [25, 26]. Recently, the molecular cytogenetic analysis of *D*. *antarctica* grown in different localities of the Maritime Antarctic and South America has been performed [23, 27]. For the other species of the genus, only chromosome numbers were determined by simple monochrome staining [28], and further comparative cytogenetic analysis of *Deschampsia* species is needed. Such studies provide important information on possible karyotypic rearrangements which occur during the speciation as well as direction of chromosomal changes in related groups. Besides, environmental stress factors can cause different changes in genome structure of plants (chromosome rearrangements, mixo- or aneuploidy) and they can also be revealed by molecular cytogenetic approaches [27, 29–33].

In the present paper, a comparative molecular cytogenetic analysis of *D*. *antarctica* (Antarctic Peninsula) and its relative species (close and distant) from different localities (*D*. *cespitosa*; *D. danthonioides* (Trin.) Munro; *D*. *flexuosa*; *D*. *elongata* (Hook.) Munro; *D*. *parvula* and *D*. *sukatschewii* (Popl.) Roshev. was firstly carried out. The karyotypes of these *Deschampsia* species were studied by DAPI-banding, fluorescence *in situ* hybridization (FISH) with 45S and 5S rDNA probes and sequential rapid genomic *in situ* hybridization (rapid GISH) with genomic DNA of *D*. *antarctica*, *D*. *cespitosa* and *D*. *flexuosa*.

## Materials and methods

### Ethics statement

This study including sample collection and experimental research conducted on these materials was according to the law on activities and environmental protection to Antarctic approved by the Ministry of Education and Science of Ukraine.

### Plant material

The seeds of *D*. *antarctica* growing under natural conditions were obtained in the course of the research Antarctic expeditions (seasons 2005–2010) organized by the National Antarctic Scientific Center of Ukraine. The seeds were collected on Rasmussen Cape (culmination of a rocky plateau: S65, S65°14.819´, W64°5.156´) located in the western coast of the Antarctic Peninsula (the vicinity of the Ukrainian Antarctic Station “Academician Vernadsky”).

The seeds of *D*. *cespitosa* (PI 314562 Moscow, Russia; W6 25808 Troms, Norway; PI 577069 Wales, UK), *D*. *danthonioides* (PI 665596 Oregon, USA; W6 39054 Washington, USA; W6 36940 Oregon, USA), *D*. *flexuosa* (PI 577075 Wales, UK; PI 422612 France) and *D*. *elongata* (PI 665545 Oregon, USA) were obtained from the germplasm collection of Western Regional Plant Introduction Station, USDA ARS NPGS, Pullman, WA, USA.

The seeds of *D*. *parvula* (661849 Weddell Island, Falkland Is.) were obtained from the germplasm collection of Seed Conservation Department, Royal Botanic Gardens, Kew, UK.

The seeds of *D*. *sukatschewii* (Popl.) Roshev. (78 Altai, Russia) were obtained from the germplasm collection of All-Russian Williams Fodder Research Institute, Moscow, Russia.

### Fixation

To produce plant material of *D*. *antarctica* in sufficient quantity, dry seeds were sterilized and germinated as described earlier [34]. The obtained aseptic plants were cultivated on B5 agar nutrient medium [35] supplemented with 0.1 mg/L α-naphthaleneacetic acid (NAA) and then the plants were *in vitro* propagated through fragmentation of the obtained root mat. Seeds of the other *Deschampsia* species were germinated in Petri dishes with moist filter paper. Then the plants were grown in a greenhouse at 15°C.

For cell cycle synchronization and accumulation of mitotic divisions, root tips were incubated in ice water for 24 hours at 0°C and then fixed in the ethanol:glacial acetic acid fixative (3:1) for 48 h at room temperature. Fixed roots were stored in the fixative at -20°C before use.

### Chromosome spread preparation

For *in situ* hybridization and DAPI staining, before chromosome spread preparation, the roots were stained in 1% acetocarmine solution in 45% acetic acid for 40 min, the tip caps with root meristem were cut on the object-plate, the meristem was macerated in a drop of 45% acetic acid, and then squashed chromosome preparations were made. The cover slips were removed after freezing, and the preparations were dehydrated and stored in 96% ethanol at -20°C before use.

### Fluorescence *in situ* hybridization

Following probes were used for FISH:

1. pTa71 containing a 9 kb long DNA sequence of common wheat encoding 18S, 5.8S and 26S rRNA genes including spacers [36];
2. pTa794 containing a 420 bp long DNA sequence of wheat containing the 5S rRNA gene and the intergenic spacer [37];

FISH assays were performed using combinations of rDNA probes labelled directly with fluorochromes SpectrumAqua or SpectrumRed (Abbott Molecular, Wiesbaden, Germany) by nick translation according to manufacturers’ protocols. FISH procedure was conducted as described previously [27, 38].

### DAPI staining

After FISH, chromosome slides were stained with 0.1 μg/ml DAPI (4',6-diamidino-2-phenylindole (DAPI, (Serva, Heidelberg, Germany)) in Vectashield mounting medium (Vector laboratories, Peterborough, UK).

### Rapid GISH procedure

Genomic DNA of *D*. *antarctica*, *D*. *cespitosa* (PI 577069) and *D*. *flexuosa* (PI 577075) was isolated from young leaves using CTAB (cetyltrimethylammonium bromide) standard protocol [39] and labelled directly with SpectrumAqua or SpectrumRed (Abbott Molecular, Wiesbaden, Germany) by nick translation according to the manufacturer’s instructions. After FISH procedures and documentation of the hybridization patterns, the chromosome slides were washed twice in 2xSSC for 10 min, dehydrated in an ethanol series (70%, 85%, 96%) for 3 min in each and air dried. Then rapid GISH procedure was conducted on the same slides as described previously [27].

### Chromosomal analysis

The slides were examined using Olympus BX61 epifluorescence microscope (Olympus, Tokyo, Japan). Images were taken with monochrome CCD camera (Cool Snap, Roper Scientific Inc., Tucson, USA) and sequentially collected in grayscale channels. Then, they were pseudocoloured and processed with Adobe Photoshop 10.0 (Adobe Systems Inc., USA) and “VideoTesT-FISH 2.1” (IstaVideotest, St Petersburg, Russia) software. At least five plants of each *Deschampsia* accession and fifteen metaphase plates from each sample were analyzed. Chromosome pairs in the species karyograms were set (considering their morphological similarities and distribution of the examined markers) according to the *D*. *antarctica* karyogram described previously [27] for simplification of the comparative karyotype analysis of different species.

## Results

### Karyotype structure

**Fig 1:**
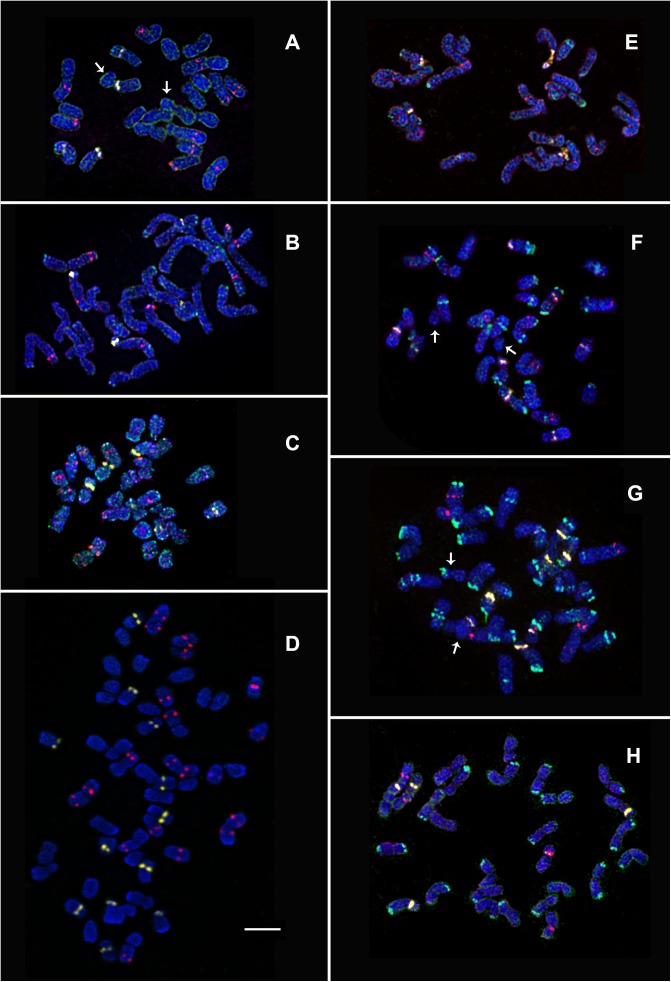
Chromosome spreads of *Deschampsia* species. (**A**) *D*. *antarctica* (2n = 26+2B), (**B**) *D*. *parvula* (2n = 26), (**C**) *D*. *cespitosa* (2n = 26), (**D**) *D*. *cespitosa* (2n = 4x = 52), (**E**) *D*. *danthonioides* (2n = 26), (**F**) *D*. *elongata* (2n = 26+2B), (**G**) *D*. *sukatschewii* (2n = 26+M+B). Merged fluorescent images after multicolour FISH with 45S (yellow) and 5S (red) rDNA and sequential rapid GISH with both genomic DNAs: *D*. *cespitosa* (green) and *D*. *flexuosa* (purple) (**A, E**, **F** and **G**); *D*. *cespitosa* (green) and *D*. *antarctica* (purple) (**H**, **B**); *D*. *antarctica* (green) and *D*. *flexuosa* (purple) (**C**). Image after FISH with 45S (yellow) and 5S (red) rDNA (**D**). Chromosomal DAPI-staining–blue. Arrows point to the marker and B chromosomes. Scale bar—5 μm.

**Fig 2:**
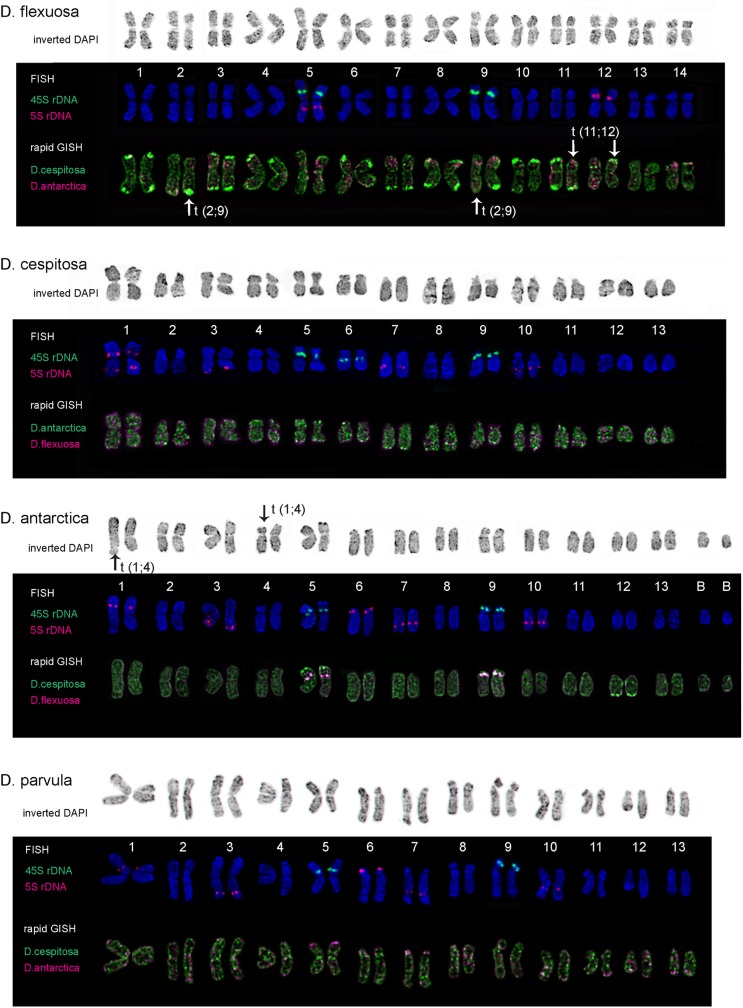
Karyotypes of *D*. *flexuosa*, *D*. *cespitosa*, *D*. *antarctica* and *D*. *parvula*. Karyograms of the metaphase plates shown in Fig 1 after DAPI-banding (inverted images), FISH with 45S and 5S rDNA, and also rapid GISH with genomic DNA of *D*. *cespitosa*, *D*. *antarctica* and *D*. *flexuosa*. The correspondent probes and their pseudo-colours are specified in the left. Arrows point to chromosome translocations. B–B chromosomes.

**Fig 3:**
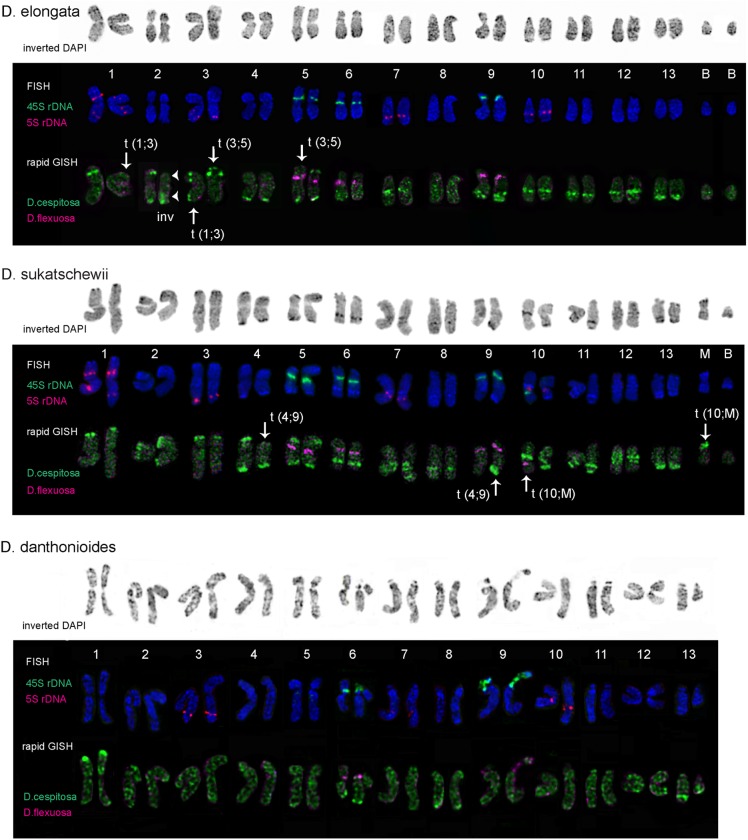
Karyotypes of specimens of *D*. *elongata*, *D*. *sukatschewii* and *D*. *danthonioides*. Karyograms of the metaphase plates shown in Fig 1 after DAPI-banding (inverted images), FISH with 45S (green) and 5S (red) rDNA, and also rapid GISH with genomic DNA of *D*. *cespitosa* (green) and *D*. *flexuosa* (red). The correspondent probes and their pseudo-colours are specified in the left. Arrows point to chromosome rearrangements. B–B chromosomes. M–a marker chromosome.

Most accessions of the studied *Deschampsia* species presented diploid karyotype with 2n = 26 chromosomes with the exception of both *D*. *flexuosa* accessions with 2n = 28 chromosomes (Figs 1–3). Besides, *D*. *cespitosa* accession PI 577069 presented a tetraploid cytotype with 2n = 52 chromosomes.

In karyotypes of D. *antarctica*, *D*. *cespitosa*, *D*. *elongata* and *D*. *sukatschewii*, 0–3 supernumerary very small chromosomes were observed together with 26 chromosomes of the basic set (A chromosomes) (Figs 1–3). These chromosomes had uncertain morphology, could be present or absent among the individuals and probably were referred to B chromosomes.

The analysis of chromosome morphology of both *D*. *flexuosa* accessions showed that its karyotype structure differed greatly from karyotypes of the other studied *Deschampsia* species and contains only metacentric and submetacentric chromosomes which were not very different in size and morphology (Figs 1 and 2).

Karyotypes of the other studied *Deschampsia* species presented common features in their structure, in particular, similar chromosome sizes and centromeric positions with one pair of metacentric chromosomes being longer than the other ones. Chromosomes in their karyotypes were subdivided into four groups: metacentric (**m**), submetacentric (**sm**), subtelocentric (**st**) and telocentric (**t**) according to chromosomes centromeric positions and morphology.

### Chromosome localization of 45S and 5S rDNA sites

In *D*. *antarctica*, FISH analysis showed that 45S rDNA loci were localized in two chromosome pairs (Fig 2): in the proximal region of the short arm of a pair of **m** chromosomes (chromosome 5) and in the distal region of the short arm of a **sm** chromosome pair (9). The secondary constriction of chromosome 9 was well-defined, and the 45S rDNA site was larger (though the satellite was smaller) compared with the other satellite (SAT) chromosome pair. We observed 5S rDNA sites on five chromosome pairs (Fig 2): in the proximal region of the short arm of the largest **m** chromosome (1), in the subtelomeric regions of the long arm of a **m** chromosome (3), in the subtelomeric regions of the short arm of a **m** chromosome (6), in the proximal regions of the long arms of a pair of **st** chromosomes (7) and a pair of **t** chromosomes (10).

In *D*. *parvula*, the pattern of 45S and 5S rDNA distribution was rather similar to that observed in *D*. *antarctica* karyotype, but there were differences in proportions of the revealed 5S rDNA. Relatively large 5S rDNA sites were found on chromosomes 3 and 6. The other 5S rDNA sites (on chromosomes 1, 7 and 10) were very small, and they were not always visualized (Fig 2).

In karyotypes of the studied *D*. *cespitosa* accessions and *D*. *elongata*, FISH analysis showed similar distribution of 45S and 5S rDNA. We detected three SAT chromosome pairs with 45S rDNA loci. Two SAT chromosome pairs (5 and 9) were very similar to those found in *D*. *antarctica* and *D*. *parvula* karyotypes; also the third SAT chromosome (6) with a large satellite was revealed (Figs 2 and 3). We observed ten 5S rDNA sites located on four chromosome pairs: in the proximal regions of the short and long arms of the largest **m** chromosome (1), in the subtelomeric regions of the long arm of a large **m** chromosome (3), in the proximal regions of the long arms of a pair of **st** chromosomes (7) and a pair of **t** chromosomes (10) (Figs 2 and 3). In karyotypes of the tetraploid *D*. *cespitosa* accession, 45S and 5S rDNA sites were localized in the similar positions and with similar proportions if compared to the diploid plants (Fig 1).

In karyotype of *D*. *sukatschewii*, multiple chromosomal rearrangements were detected. In particular, in each metaphase cell of the studied accession, we observed co-localization of the 5S and 45S rDNA sites in one of the homologs of chromosome 10 (the long arm) and also a marker chromosome (M) which probably resulted from a translocation between chromosome 10 and an unknown supernumerary chromosome having a 45S rDNA site (Figs 1 and 3). Nevertheless, the pattern of chromosomal distribution of 45S and 5S rDNA loci was similar (with the variation mentioned above) to that described for *D*. *cespitosa* and *D*. *elongata*.

In karyotypes of the studied *D*. *danthonioides* accessions, FISH analysis revealed two SAT chromosomes with 45S rDNA loci (chromosomes 6 and 9) (Fig 3). In some karyotypes, minor 45S rDNA sites were revealed in the proximal region of chromosome 5. In most cases, 5S rDNA sites were observed on chromosomes 3 and 10. In some karyotypes, very small 5S rDNA site was also detected on chromosome 7 (Fig 3). The revealed 45S and 5S rDNA sites were located in the similar positions if compared to the other studied *Deschampsia* species (with exception of *D*. *flexuosa*).

The pattern of chromosomal localization of 45S and 5S rDNA loci in karyotypes of both *D*. *flexuosa* accessions differed from the patterns of rDNA distribution in karyotypes of the other studied *Deschampsia* species. 45S rDNA loci were detected in the secondary construction regions (the short arms) of two pairs of SAT chromosomes. Also, two sites of 5S rDNA were detected. One 5S rDNA locus was localized in the proximal region of the long arm of one SAT chromosome pair. The second 5S rDNA site was revealed in the pericentromeric region of the short arm of a pair of metacentric chromosomes (Fig 2).

### Chromosomal markers revealed by rapid GISH

Rapid GISH procedure with total genomic DNA of *D*. *antarctica* as a DNA probe was performed on chromosomes of *D*. *cespitosa*, *D*. *parvula* and *D*. *flexuosa*. Multiple rapid GISH markers (in different positions) were detected on chromosomes of the studied *D*. *cespitosa* accessions (Fig 2). The dispersed hybridization signals were observed along chromosomes of *D*. *parvula* and both *D*. *flexuosa* accessions (Fig 2).

Rapid GISH procedure with total genomic DNA of *D*. *cespitosa* (PI 577069) as a DNA probe was performed on chromosomes of *D*. *antarctica*, *D*. *danthonioides*, *D*. *flexuosa*, *D*. *elongata*, *D*. *parvula* and *D*. s*ukatschewii*. As a result, on chromosomes of *D*. *elongata*, *D*. s*ukatschewii* and both *D*. *flexuosa* accessions, multiple clustered hybridization signals (rapid GISH markers) were revealed in different positions (Figs 1–3). Besides, large rapid GISH markers were detected in subtelomeric regions of the short arms of chromosomes 5, 9 and the long arm of chromosome 12 of *D*. *antarctica* (Fig 2); in the subtelomeric regions of the short arm of chromosomes 1 and the long arms of chromosomes 6, 8, 12 and 13 in karyotypes of the studied *D*. *danthonioides* accessions (Fig 3). Also, the dispersed hybridization signals were observed along chromosomes of *D*. *parvula* (Fig 2).

Rapid GISH procedure with total genomic DNA of *D*. *flexuosa* (PI 577075) as a DNA probe was carried out on chromosomes of *D*. *antarctica*, *D*. *cespitosa*, *D*. *danthonioides*, *D*. *elongata*, *D*. *parvula*, and *D*. *sukatschewii*. In all the studied accessions, weak dispersed hybridization signals were detected along the chromosomes. Also, in karyotypes of *D*. *antarctica*, *D*. *elongata*, *D*. *sukatschewii* and the studied *D*. *danthonioides* accessions, large rapid GISH markers were found in the secondary constriction regions (NORs) of SAT chromosomes (Figs 1–3).

### DAPI-banding analysis

After FISH procedures, chromosome staining with DAPI revealed banding patterns (DAPI-banding). Analysis of distribution of DAPI-bands in karyotypes of the studied *Deschampsia* species showed that the most intense bands were located in the pericentromeric and subtelomeric regions of chromosomes, while a number of small and also faint inconsistent bands were detected in the interstitial chromosome regions. Visual analysis showed that chromosomal DAPI-banding patterns were specific to the studied species as variations in size, number and localization of the bands was observed (Figs 2 and 3). Chromosomal distribution of DAPI-bands within the studied accessions of the species was similar though variations in size and intensity of DAPI-bands were also observed. The largest DAPI-bands were observed on the chromosomes of *D*. *sukatschewii* specimen (Fig 3). B chromosomes, found in karyotypes of *D*. *antarctica*, *D*. *cespitosa*, *D*. *sukatschewii* and *D*. *elongata*, possessed distinct DAPI -bands in their subtelomeric regions (Figs 2 and 3).

### Karyotype analysis

Based on chromosomal morphology, DAPI-banding patterns, localization of 45S and 5S rDNA and rapid GISH markers, chromosomes in karyotypes of the studied *Deschampsia* species were identified and karyograms were constructed (Figs 2 and 3). We note that patterns of distribution of the examined markers were similar in karyotypes of the studied species accessions; accordingly, one karyogram of each species is presented.

The comparison of patterns of distribution of the examined molecular cytogenetic markers (DAPI-bands, 45S and 5S rDNA, and also rapid GISH markers) allowed us to reveal chromosomal rearrangements in karyotypes of *D*. *cespitosa*, *D*. *danthonioides*, *D*. *flexuosa*, *D*. *elongata* and *D*. *sukatschewii* (detailed in Figs 2 and 3).

## Discussion

Most *Deschampsia* species were shown to have a basic chromosome number х = 13 with the exception of *D*. *setacea* (2n = 2х = 14), *D*. *atropurpurea* (2n = 2х = 14) and *D*. *flexuosa* (2n = 4х = 28) which present a typical for cereals basic number of x = 7 [28, 31]. In the present study, the chromosome numbers were verified for normal diploid karyotypes of *D*. *antarctica*, *D*. *cespitosa*, *D*. *danthonioides*, *D*. *flexuosa*, *D*. *elongata*, *D*. *parvula* and *D*. *sukatschewii*.

We also detected different ploidy levels in *D*. *cespitosa* as both diploid (2n = 26) and tetraploid (2n = 52) *D*. *cespitosa* cytotypes were revealed. The results reported here are in good agreement with the data on different ploidy levels (2n = 26; 3n-39; 4n = 52) as well as inter- and intra-individual variability in chromosome number (2n = 15–28) observed in several taxa of *D*. *cespitosa* complex [1, 31]. Polyploidy is widespread in the grasses [40]. It is considered to be an important driving force in plant evolution [41], and several genomic duplication events were postulated in the evolution of the family *Poaceae* [42]. The adaptive significance of the variability in ploidy level found in some *Deschampsia* species is still unclear. It might be related to ability of polyploids to colonize larger geographic ranges and/or occur in more habitats than related diploids [41]. This is probably caused by greater genetic and biochemical variability of polyploid genome [43].

In *D*. *antarctica* accession collected on Rasmussen Cape, we revealed only a diploid cytotype. However, the presence of polyploid and mixoploid populations of *D*. *antarctica* in other localities of the Antarctic regions was previously described [23, 27–28]. Polyploidy and hybridization processes were considered to play an important role in the evolution of the genome of *D*. *antarctica* which might be related to high tolerance and genomic plasticity of this species [23, 27].

B chromosomes were described in karyotypes of different grass species [44–45] including some species of *D*. *cespitosa* complex [31] and also in *D*. *antarctica* located in the Maritime Antarctic [27]. It was shown that Bs can be present or absent among individuals within the same species population due to their dispensable nature [46–47]. There are also data suggesting that Bs can be spontaneously generated in response to the new genome conditions following interspecific hybridization [33, 48]. The functional role of the observed Bs is still uncertain, but the correlation between the appearance of Bs in a karyotype and environmental conditions was found [49–51]. Besides, they are mostly observed in karyotypes of the species having wide distribution area with various environmental conditions [52]. In agreement with these observations, we found Bs in karyotypes of *D*. *antarctica*, *D*. *cespitosa*, *D*. *elongata* and *D*. *sukatschewii* which can grow in different edaphoclimatic conditions including extreme habitats [31].

The cytogenetic analysis performed in the present study, indicated karyotypic similarities among the studied *Deschampsia* species with the exception of the most distantly related *D*. *flexuosa*. Chromosomes of *D*. *flexuosa* were not very different in size and centromere positions whereas the karyotypes of the other studied *Deschampsia* species contained **m**, **sm**, **st** and **t** chromosomes of different sizes with one pair of **m** chromosomes being longer than the others. Our results are in agreement with earlier reported data on the karyotypes of few *Deschampsia* species [1, 25–28]. This long metacentric chromosome was suggested to be a result of a translocation (or a centric fusion) between two acrocentric chromosomes, giving rise to the unusual for cereals chromosome number 2n = 26 [1]. The intercalary signal of telomere repeats in that long chromosome previously revealed by FISH [27] confirms indirectly this assumption as intercalary sites of telomere repeats are considered to be the remnants of ancestral chromosomal rearrangements occurred during the evolution [53].

After FISH, DAPI staining displays regions of AT-rich heterochromatin as strongly stained bands (DAPI-banding patterns) in karyotypes of vascular plants [54] including *D*. *antarctica* [27]. Accordingly, in this study DAPI-banding patterns were also used for chromosome analysis of *Deschampsia* species. The results of FISH with 45S and 5S rDNA as well as chromosomal distribution of DAPI-bands in *D*. *antarctica* collected on Rasmussen Cape were in good agreement with karyotypic data previously described for *D*. *antarctica* specimens grown in several localities of Maritime Antarctic and South America [23, 27].

Comparison of *D*. *antarctica* karyotype with the other studied *Deschampsia* species revealed basic karyotypic similarities though structural peculiarities among these species were also found. Particularly, in the studied specimens of *D*. *antarctica* and closely related *D*. *parvula* from Falkland Islands, which are a biogeographical part of the mild Antarctic zone with the closest to the Antarctic habitats (compared to the other species), 45S and 5S rDNA sites were distributed in similar chromosomal positions. However, in *D*. *parvula*, some 5S rDNA sites were very small and not always detected. It might be related to chromosomal redistribution of 5S rDNA occurred during the speciation process. Nevertheless, since *D*. *parvula* karyotype was studied for the first time, it could be a manifestation of karyotypic polymorphism of the studied population, and other specimens from different localities should be studied. Moreover, these species were rather similar in content of AT-heterochromatin though differences in the number, size and position of some DAPI-bands were detected.

In three studied accessions of *D*. *cespitosa*, chromosomal distribution of 45S and 5S rDNA loci differed from that found in *D*. *antarctica*. In diploid karyotypes of *D*. *cespitosa*, we found three SAT chromosome pairs (5, 6 and 9) bearing 45S rDNA: two of them (with proximal and distal 45S rDNA sites) were similar to those found in *D*. *antarctica* and another SAT chromosome pair possesses a proximal 45S rDNA site. Similar to *D*. *antarctica*, ten 5S rDNA loci were revealed in karyotypes of *D*. *cespitosa* but they were localized in four chromosome pairs as the longest chromosome 1 possesses two 5S rDNA loci in both arms. These observations corresponded to karyotypic data described earlier for *D*. *cespitosa* [25]. However, since only limited European accessions of *D*. *cespitosa* have been studied to date, further molecular cytogenetic analyses of *D*. *cespitosa* collected from different localities (and environmental conditions including extreme habitats) are needed in order to investigate the possible chromosome changes in distant populations.

In karyotypes of *D*. *elongata* and *D*. *sukatschewii*, the number and chromosomal localization of 45S and 5S rDNA (characterized for the first time for both species) were very similar to *D*. *cespitosa* despite multiple chromosomal rearrangements found in *D*. *sukatschewii*. Interestingly, in the studied *D*. *sukatschewii* accession, a chromosome rearrangement involving rDNA loci (co-localized 5S and 45S rDNA sites detected in one of the homologous chromosomes 10) was observed. It is clearly impossible for plants to go through meiosis with such an unbalanced karyotype, and the appearance and maintenance of these chromosome rearrangements in the population could be due to the capacity of *Deschampsia* species to reproduce by self-fertilization and/or asexual (vegetative or apomictic) propagation [24, 31, 55–58]. The chromosomal DAPI-banding patterns of *D*. *cespitosa*, *D*. *elongata* and *D*. *sukatschewii* were also found to follow the common pattern though species differences in the number, size and position of some bands were observed. Our results agree with early reported molecular phylogenetic data (the analysis of ITS and plastid trnL intron sequences) which showed close relationships between these species [2, 22–24].

In karyotypes of the studied *D*. *danthonioides* accessions, FISH analysis (performed for the first time) detected only two 45S rDNA loci. It was fewer if compared to *D*. *cespitosa* karyotype with three sites and similar to *D*. *antarctica* karyotype. However, we observed the minor 45S rDNA sites in the proximal region of the short arm of chromosome 5 which might indicate the direction of genome changes occurred during evolution in *D*. *danthonioides*. Besides, fewer 5S rDNA sites were revealed compared to *D*. *cespitosa* and *D*. *antarctica* though they were located in the similar positions if compared to the other studied *Deschampsia* species (with exception of *D*. *flexuosa*). Chromosomal DAPI-banding patterns in *D*. *danthonioides* also differ from those found in karyotypes of both *D*. *antarctica* and *D*. *cespitosa* though early reported molecular phylogenetic data showed that *D*. *danthonioides* is closely related to *D*. *cespitosa* [2, 22–24].

In karyotypes of both studied *D*. *flexuosa* accessions, the number and localization of 45S and 5S rDNA sites (characterized for the first time) differed significantly from those detected in the other studied *Deschampsia* species. Interestingly, we observed one chromosome pair possessed both 45S (in the short arm) and 5S rDNA loci (in the long arm) though, in several genera and groups of closely related species including *Deschampsia*, *Avena*, *Holcus* and *Agrostis*, investigated to date, 5S rDNA is considered to localize exclusively in chromosomes without NORS [59]. The localization of 5S and 45S rDNA sites in one chromosome pair could be related to chromosomal rearrangements occurred in *D*. *flexuosa* during the speciation processes.

It has been previously shown that FISH with the repetitive DNA probes (pSc119.2, pAs1 and GAA-microsatellite sequence), specific for chromosomes of most members of the family *Poaceae*, was not informative for *D*. *antarctica* karyotype [27]. Accordingly, for comparative chromosome analysis of the studied *Deschampsia* species, we applied a rapid GISH procedure with labelled genomic DNA of *D*. *antarctica*, closely related *D*. *cespitosa* and distantly related *D*. *flexuosa* in different combinations. This approach allowed us to reveal homologous highly repeated DNA sequences between genomes of the related species and presented additional markers for chromosomal identification. The analysis of patterns of chromosomal distribution of the examined markers indicated a lot of chromosomal rearrangements in karyotypes of the studied *Deschampsia* species which indicate that process of genome evolution of the genus *Deschampsia* could include chromosomal reorganization (chromosome interchanges, inversions, translocations) of the initial parental genomes since the presence of numerous chromosomal rearrangements in plant genomes are considered to be speciation-related events [60–61].

Rapid GISH procedure with genomic DNA of *D*. *cespitosa* as a DNA probe revealed three clustered signals (markers) on chromosomes of *D*. *antarctica* confirming the results obtained previously with genomic DNA of the other accession of *D*. *cespitosa* [27]. However, in karyotypes of closely related *D*. *parvula*, rapid GISH with this probe detected only two markers indicating some differences between genomes of *D*. *antarctica* and *D*. *parvula*.

On chromosomes of *D*. *elongata* and *D*. s*ukatschewii*, rapid GISH with genomic DNA of *D*. *cespitosa* revealed multiple rapid GISH markers which were similarly distributed. These findings confirm the close relationship among the three species. On chromosomes of *D*. *danthonioides*, rapid GISH with this probe detected only four clustered hybridization signals in different positions compared to *D*. *elongata* and *D*. s*ukatschewii* indicating the presence of structural differences between the genomes of *D*. *danthonioides* and the other three species. These cytogenetic data are in agreements with our results on FISH with 5S and 45S rDNA as well as molecular phylogenetic data described previously for these species [22, 23].

According to the analysis of ITS tree, *D*. *flexuosa* is now regarded a better suited to the genus *Avenella* [22]. Additionally, its karyotype differed significantly from the rest of the species studied here. Meanwhile, *D*. *flexuosa* is very similar morphologically to *D*. *cespitosa*, and also the genomic DNA of both *D*. *antarctica* and *D*. *cespitosa* as DNA probes detected multiple rapid GISH markers (in different positions) on chromosomes of *D*. *flexuosa* indicating the presence of a great number of homologous highly repeated DNA sequences in their genomes. These observations show that *D*. *flexuosa* is related to the species of *D*. *cespitosa* complex, and it might take an intermediate position between the two allied genera *Deschampsia* and *Avenella*.

Interestingly, the reverse GISH procedure (e.g., using the genomic DNA of *D*. *flexuosa* as a probe) presented dispersed hybridization signals on chromosomes of *D*. *antarctica* and *D*. *cespitosa* indicating that the homologous highly repeated DNA sequences were distributed differently in their genomes. Also, weak dispersed signals of this probe were revealed on chromosomes of the other studied *Deschampsia* species. Consequently, during the speciation process clustering of some common repeats occurred in *D*. *flexuosa* genome whereas in genomes of the other studied *Deschampsia* species those repeats remained unclustered.

Thus, though the molecular cytogenetic analysis showed similar features in the karyotype structure of most studied *Deschampsia* species, significant interspecific variability in chromosome localization of the examined markers was found. Based on the analysis of chromosome morphology and patterns of distribution of the examined markers, the studied *Deschampsia* species can be divided into four karyological groups (with some interspecific chromosomal differences inside group 1 and 2): 1) *D*. *antarctica* and *D*. *parvula*; 2) *D*. *cespitosa*, *D*. *elongata* and *D*. *sukatschewii*; 3) *D*. *danthonioides*; 4) *D*. *flexuosa*.

## Conclusions

Though polyploidy and hybridization play an important role in speciation, our findings show that the processes of genome reorganization of *Deschampsia* species during evolution could involve different chromosomal rearrangements.

The unique *D*. *antarctica* karyotype has experienced a significant chromosome rearrangements (compared to *D*. *cespitosa* complex). The karyotype of *D*. *parvula* from Falkland Islands with the closest to the Antarctic habitats is similar (with some variations) to *D*. *antarctica* and also differs from the core of the genus. The detected differences in karyotype of the remote *D*. *danthonioides* (the North America) indicate the need to perform a comparative molecular cytogenetic analysis of *D*. *cespitosa* populations from different habitats, in particular, from different continents or from North and South hemispheres. *D*. *flexuosa* karyotype differs significantly from the rest of the studied species; however, the presence of common DNA repeats show that this species is related to *D*. *cespitosa* complex or they have the common ancestral species. The obtained results will help clarify the relationships within the genus *Deschampsia*, and can be a basis for the further genetic and biotechnological studies as well as for selection of plants tolerant to the extreme habitats.
